# 2-Benzoyl­amino-*N*-[5-(4-bromo­phen­yl)-1,3,4-thia­diazol-2-yl]ethanamide

**DOI:** 10.1107/S160053680900124X

**Published:** 2009-01-17

**Authors:** Hui-Ming Huang, Shi-Yuan Luo, Shao-Hua Li, Cheng-Mei Liu, Guo-Gang Tu

**Affiliations:** a State Key Laboratory of Food Science and Technology, Nanchang University, 330047 Nanchang, JiangXi, People’s Republic of China; b Department of Pharmacy, NanChang University Medical College, 330006 Nanchang, JiangXi, People’s Republic of China

## Abstract

In the structure of the title compound, C_17_H_13_BrN_4_O_2_S, the dihedral angle between the two benzene rings is 38.5 (1)°; the angle between the 4-bromo­benzene and thia­diazole rings is 1.3 (1)°. The conformations of the N—H and C=O bonds are *anti *with respect to each other. The structure displays inter­molecular N—H⋯O and C—H⋯O hydrogen bonding, with both interactions leading to inversion dimers.

## Related literature

For 1,3,4-thia­diazole scaffold compounds and their biological activity, see: Tu *et al.* (2008 ▶). For the synthesis, see: Foroumadi *et al.* (1999 ▶); Levy & Palmer (1942 ▶); Song *et al.* (1992 ▶). For related structures, see: Gowda *et al.* (2008 ▶); Li, Huang *et al.* (2008 ▶); Li, Li *et al.* (2008 ▶).
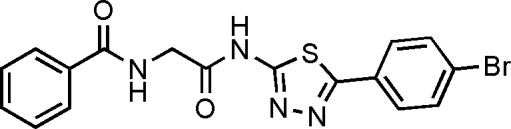


## Experimental

### 

#### Crystal data

C_17_H_13_BrN_4_O_2_S
*M* *_r_* = 417.28
Triclinic, 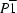

*a* = 4.020 (4) Å
*b* = 13.706 (9) Å
*c* = 16.210 (5) Å
α = 113.334 (17)°
β = 94.018 (19)°
γ = 92.78 (2)°
*V* = 815.2 (10) Å^3^
*Z* = 2
Mo *K*α radiation
μ = 2.67 mm^−1^
*T* = 298 (2) K
0.54 × 0.17 × 0.04 mm

#### Data collection

Bruker X8 APEXII diffractometer
Absorption correction: multi-scan (*SADABS*; Bruker, 2008 ▶) *T*_min_ = 0.591, *T*_max_ = 0.914
4633 measured reflections
2592 independent reflections
1097 reflections with *I* > 2σ(*I*)
*R*_int_ = 0.082

#### Refinement

*R*[*F* ^2^ > 2σ(*F* ^2^)] = 0.109
*wR*(*F* ^2^) = 0.281
*S* = 0.82
2592 reflections
227 parameters
6 restraints
H-atom parameters constrained
Δρ_max_ = 1.67 e Å^−3^
Δρ_min_ = −1.40 e Å^−3^

### 

Data collection: *APEX2* (Bruker, 2004 ▶); cell refinement: *SAINT* (Bruker, 2004 ▶); data reduction: *SAINT*; program(s) used to solve structure: *SHELXS97* (Sheldrick, 2008 ▶); program(s) used to refine structure: *SHELXL97* (Sheldrick, 2008 ▶); molecular graphics: *APEX2* (Bruker, 2004 ▶); software used to prepare material for publication: *APEX2* (Bruker, 2004 ▶) and *publCIF* (Westrip, 2009 ▶).

## Supplementary Material

Crystal structure: contains datablocks I, global. DOI: 10.1107/S160053680900124X/wn2304sup1.cif

Structure factors: contains datablocks I. DOI: 10.1107/S160053680900124X/wn2304Isup2.hkl

Additional supplementary materials:  crystallographic information; 3D view; checkCIF report

## Figures and Tables

**Table 1:** Hydrogen-bond geometry (Å, °)

*D*—H⋯*A*	*D*—H	H⋯*A*	*D*⋯*A*	*D*—H⋯*A*
C16—H16*A*⋯O2^i^	0.93	2.49	3.400 (5)	168
N2—H2*A*⋯O1^ii^	0.86	1.99	2.835 (5)	167

Symmetry codes: (i) 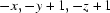; (ii) 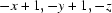.

**Fig. 1.**
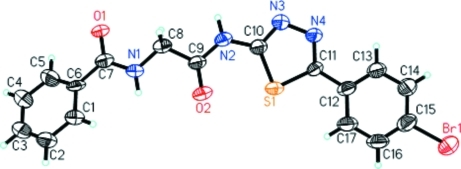
Molecular structure of the title compound. Displacement ellipsoids are drawn at the 50% probability level. Hydrogen atoms are shown as spheres of arbitrary radius.

**Fig. 2.**
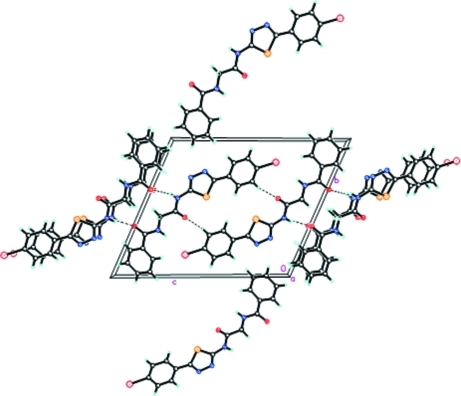
The crystal packing of the title compound, viewed along the a axis with hydrogen bonds drawn as dashed lines.

## supplementary crystallographic information

### Comment

In our previous work, 1,3,4-thiadiazole scaffold compounds and their biological activity have been studied (Tu *et al.*, 2008). In view of the importance of these organic materials, the title compound (Fig. 1) was synthesized (Foroumadi *et al.*, 1999; Levy & Palmer 1942; Song *et al.*, 1992) and its crystal structure is reported here.

In the structure of the title compound, C_17_H_13_BrN_4_O_2_S, the dihedral angle between the *p*-bromobenzene and thiadiazole rings is 1.3 (1)°; the angle between the two benzene rings is 38.5 (1)°. The conformations of the N—H and C═O bonds are *anti* with respect to each other. Bond lengths and angles are in normal ranges and comparable to those in related structures (Gowda *et al.*, 2008; Li, Huang *et al.*, 2008; Li, Li *et al.*, 2008). In the crystal structure, molecules are linked through intermolecular C—H···O and N—H···O hydrogen bonds, forming a three-dimensional network (Table 1, Figure 2).

### Experimental

*N,N*-Dicyclohexylcarbodiimide (5.7 mmol) was added to a cooled solution of *N*-benzoylglycine (5.6 mmol) and *N*-hydroxysuccinimide (5.6 mmol) in freshly distilled dioxane (30 ml). The reaction mixture was stirred overnight at room temperature. The insoluble material was filtered off and washed with cold dioxane. 2-Amino-5-(4-bromophenyl)-1,3,4-thiadiazole (5.5 mmol) was added to the filtrate and the reaction mixture was stirred for 48 h at room temperature. The solvent was removed under reduced pressure. The residue was dissolved in EtOAc and the insoluble material was filtered off. The filtrate was washed successively with saturated Na_2_CO_3_ solution (20 ml, *x* 3), water (20 ml, *x* 1), 0.1 *M* HCl (20 ml, *x* 3) and water (20 ml, *x* 1). The organic layer evaporated *in vacuo*, and the residue was recrystallized from methanol. Colorless block-shaped single crystals of the title compound suitable for X-ray diffraction analysis precipitated after several days.Yield: 37.0%; mp: 271–273°C.

### Refinement

H atoms were positioned geometrically and refined using a riding model; Csp^2^—H = 0.93 Å, Csp^3^—H = 0.97 Å and N—H = 0.86 Å; *U*_ĩso_(H) = 1.2 *U*_eq_(C,N). We made several attempts to obtain better quality data for this structure. However, due to poor crystal quality and possible disorder, the *R* and *wR* values are high. The maximum residual electron density occurs 1.23 Å from atom Br1, and the minimum residual electron density is located 1.29 Å from atom Br1.

### Crystal data

**Table:**

C_17_H_13_BrN_4_O_2_S	*Z* = 2
*M**_r_* = 417.28	*F*(000) = 420.0
Triclinic, *P*1	*D*_x_ = 1.700 Mg m^−^^3^
Hall symbol: -P 1	Mo *K*α radiation, λ = 0.71073 Å
*a* = 4.020 (4) Å	Cell parameters from 1179 reflections
*b* = 13.706 (9) Å	θ = 2.6–23.7°
*c* = 16.210 (5) Å	µ = 2.67 mm^−^^1^
α = 113.334 (17)°	*T* = 298 K
β = 94.018 (19)°	Block, colourless
γ = 92.78 (2)°	0.54 × 0.17 × 0.04 mm
*V* = 815.2 (10) Å^3^	

### Data collection

**Table:**

Bruker X8 APEXII diffractometer	2592 independent reflections
Radiation source: fine-focus sealed tube	1097 reflections with *I* > 2σ(*I*)
graphite	*R*_int_ = 0.082
φ and ω scans	θ_max_ = 25.0°, θ_min_ = 2.5°
Absorption correction: multi-scan (*SADABS*; Bruker, 2008)	*h* = −4→4
*T*_min_ = 0.591, *T*_max_ = 0.914	*k* = −16→16
4633 measured reflections	*l* = −19→19

### Refinement

**Table:**

Refinement on *F*^2^	Secondary atom site location: difference Fourier map
Least-squares matrix: full	Hydrogen site location: inferred from neighbouring sites
*R*[*F*^2^ > 2σ(*F*^2^)] = 0.109	H-atom parameters constrained
*wR*(*F*^2^) = 0.281	*w* = 1/[σ^2^(*F*_o_^2^) + (0.1951*P*)^2^] where *P* = (*F*_o_^2^ + 2*F*_c_^2^)/3
*S* = 0.82	(Δ/σ)_max_ = 0.019
2592 reflections	Δρ_max_ = 1.67 e Å^−^^3^
227 parameters	Δρ_min_ = −1.39 e Å^−^^3^
6 restraints	Extinction correction: *SHELXL97* (Sheldrick, 2008), Fc^*^=kFc[1+0.001xFc^2^λ^3^/sin(2θ)]^-1/4^
Primary atom site location: structure-invariant direct methods	Extinction coefficient: 0.022 (2)

### Special details

**Table:**

Experimental. ^1^H-NMR (DMSO-*d6*): δ 4.24–4.25(d, *J*=5.08 Hz, 2H), 7.49–7.57 (m, 3H),7.73–7.75 (d, *J*=8.04 Hz, 2H), 7.89–7.91(t, *J*=3.60 Hz, 4H), 9.01 (s, 1H),12.91 (s, 1H). ESI-MS: *m/z* [*M*+H]^+^ 417.3.
Geometry. All e.s.d.'s (except the e.s.d. in the dihedral angle between two l.s. planes) are estimated using the full covariance matrix. The cell e.s.d.'s are taken into account individually in the estimation of e.s.d.'s in distances, angles and torsion angles; correlations between e.s.d.'s in cell parameters are only used when they are defined by crystal symmetry. An approximate (isotropic) treatment of cell e.s.d.'s is used for estimating e.s.d.'s involving l.s. planes.
Refinement. Refinement of *F*^2^ against ALL reflections. The weighted *R*-factor *wR* and goodness of fit *S* are based on *F*^2^, conventional *R*-factors *R* are based on *F*, with *F* set to zero for negative *F*^2^. The threshold expression of *F*^2^ > σ(*F*^2^) is used only for calculating *R*-factors(gt) *etc*. and is not relevant to the choice of reflections for refinement. *R*-factors based on *F*^2^ are statistically about twice as large as those based on *F*, and *R*- factors based on ALL data will be even larger.

### Fractional atomic coordinates and isotropic or equivalent isotropic displacement parameters (Å^2^)

**Table:**

	*x*	*y*	*z*	*U*_iso_*/*U*_eq_	
Br1	0.19570 (14)	0.16142 (4)	0.64242 (3)	0.07526 (19)	
S1	0.3284 (3)	0.41677 (8)	0.33059 (7)	0.0550 (4)	
O1	0.2257 (8)	0.6333 (2)	−0.00717 (17)	0.0636 (9)	
O2	0.2253 (9)	0.5689 (2)	0.25867 (18)	0.0687 (8)	
N1	0.2711 (9)	0.6635 (2)	0.1388 (2)	0.0551 (12)	
H1A	0.2352	0.7034	0.1931	0.066*	
N3	0.5949 (10)	0.2787 (3)	0.1998 (2)	0.0621 (13)	
N4	0.5645 (10)	0.2351 (2)	0.2632 (2)	0.0597 (8)	
N2	0.4834 (9)	0.4276 (2)	0.1728 (2)	0.0539 (8)	
H2A	0.5726	0.3992	0.1229	0.065*	
C4	−0.2078 (13)	0.9136 (3)	0.0368 (3)	0.0711 (18)	
H4B	−0.3108	0.9254	−0.0112	0.085*	
C5	−0.0907 (12)	0.8176 (3)	0.0241 (3)	0.0610 (16)	
H5A	−0.1231	0.7628	−0.0332	0.073*	
C10	0.4756 (11)	0.3703 (3)	0.2263 (3)	0.0512 (11)	
C16	0.1797 (12)	0.3039 (3)	0.5556 (3)	0.0632 (17)	
H16A	0.0838	0.3486	0.6061	0.076*	
C15	0.2691 (13)	0.2067 (3)	0.5481 (3)	0.0603 (10)	
C12	0.3711 (10)	0.2674 (3)	0.4098 (2)	0.0471 (9)	
C14	0.4058 (11)	0.1370 (3)	0.4729 (3)	0.0598 (11)	
H14A	0.4596	0.0701	0.4688	0.072*	
C17	0.2354 (12)	0.3345 (3)	0.4857 (3)	0.0587 (11)	
H17A	0.1807	0.4014	0.4900	0.070*	
C8	0.4114 (12)	0.5638 (3)	0.1200 (3)	0.0560 (15)	
H8A	0.6486	0.5723	0.1144	0.067*	
H8B	0.3068	0.5116	0.0627	0.067*	
C9	0.3647 (11)	0.5237 (3)	0.1913 (2)	0.0475 (10)	
C11	0.4297 (11)	0.2963 (3)	0.3341 (2)	0.0483 (10)	
C13	0.4598 (12)	0.1698 (3)	0.4041 (3)	0.0601 (16)	
H13A	0.5571	0.1253	0.3537	0.072*	
C7	0.1970 (11)	0.6938 (3)	0.0722 (3)	0.0521 (13)	
C6	0.0730 (11)	0.7991 (3)	0.0926 (2)	0.0489 (14)	
C2	−0.0154 (12)	0.9776 (3)	0.1919 (3)	0.0601 (16)	
H2B	0.0074	1.0320	0.2494	0.072*	
C3	−0.1698 (12)	0.9930 (3)	0.1230 (3)	0.0643 (17)	
H3B	−0.2533	1.0585	0.1332	0.077*	
C1	0.1113 (12)	0.8805 (3)	0.1780 (3)	0.0555 (15)	
H1B	0.2216	0.8701	0.2259	0.067*	

### Atomic displacement parameters (Å^2^)

**Table:**

	*U*^11^	*U*^22^	*U*^33^	*U*^12^	*U*^13^	*U*^23^
Br1	0.0941 (4)	0.0888 (3)	0.0604 (2)	0.0226 (3)	0.0361 (2)	0.04206 (19)
S1	0.0804 (8)	0.0441 (5)	0.0406 (5)	0.0193 (5)	0.0286 (5)	0.0118 (4)
O1	0.106 (2)	0.0501 (14)	0.0414 (6)	0.0279 (14)	0.0433 (10)	0.0174 (7)
O2	0.1043 (12)	0.0616 (16)	0.0469 (7)	0.0256 (13)	0.0439 (6)	0.0207 (9)
N1	0.082 (3)	0.0452 (17)	0.0435 (16)	0.0255 (16)	0.0332 (18)	0.0165 (13)
N3	0.099 (3)	0.0488 (17)	0.0448 (17)	0.0288 (18)	0.0270 (19)	0.0193 (13)
N4	0.1059 (15)	0.0386 (15)	0.0399 (8)	0.0252 (12)	0.0297 (7)	0.0154 (8)
N2	0.075 (2)	0.0474 (5)	0.0474 (5)	0.0157 (15)	0.0353 (16)	0.0212 (3)
C4	0.097 (4)	0.049 (2)	0.070 (3)	0.025 (2)	0.022 (3)	0.022 (2)
C5	0.080 (3)	0.042 (2)	0.053 (2)	0.012 (2)	0.009 (2)	0.0102 (18)
C10	0.073 (3)	0.0390 (7)	0.0442 (7)	0.0091 (19)	0.024 (2)	0.0156 (4)
C16	0.080 (3)	0.054 (2)	0.051 (2)	0.012 (2)	0.034 (2)	0.0102 (19)
C15	0.0825 (16)	0.0564 (19)	0.0442 (9)	−0.0018 (12)	0.0194 (8)	0.0211 (8)
C12	0.0546 (15)	0.0498 (18)	0.0351 (9)	0.0036 (12)	0.0172 (7)	0.0131 (8)
C14	0.066 (2)	0.066 (2)	0.0600 (12)	0.0136 (14)	0.0282 (10)	0.0337 (10)
C17	0.078 (2)	0.050 (2)	0.0448 (11)	0.0131 (15)	0.0261 (10)	0.0109 (10)
C8	0.064 (3)	0.061 (2)	0.053 (2)	0.026 (2)	0.035 (2)	0.0269 (16)
C9	0.0644 (16)	0.041 (2)	0.0353 (8)	0.0082 (16)	0.0210 (7)	0.0104 (12)
C11	0.0753 (18)	0.0407 (18)	0.0336 (10)	0.0106 (14)	0.0223 (9)	0.0165 (9)
C13	0.094 (3)	0.042 (2)	0.046 (2)	0.010 (2)	0.030 (2)	0.0159 (16)
C7	0.071 (3)	0.047 (2)	0.0344 (7)	0.0104 (19)	0.0185 (14)	0.0105 (9)
C6	0.061 (3)	0.047 (2)	0.0339 (18)	0.0118 (19)	0.030 (2)	0.0067 (16)
C2	0.078 (3)	0.043 (2)	0.050 (2)	0.009 (2)	0.019 (2)	0.0062 (18)
C3	0.080 (3)	0.052 (2)	0.065 (3)	0.031 (2)	0.025 (3)	0.0211 (19)
C1	0.071 (3)	0.047 (2)	0.046 (2)	0.016 (2)	0.023 (2)	0.0120 (17)

### Geometric parameters (Å, °)

**Table:**

Br1—C15	1.898 (5)	C16—C17	1.382 (7)
S1—C10	1.714 (4)	C16—H16A	0.9300
S1—C11	1.741 (5)	C15—C14	1.387 (6)
O1—C7	1.242 (4)	C12—C13	1.370 (6)
O2—C9	1.214 (5)	C12—C17	1.380 (5)
N1—C7	1.323 (6)	C12—C11	1.461 (6)
N1—C8	1.430 (5)	C14—C13	1.383 (7)
N1—H1A	0.8600	C14—H14A	0.9300
N3—C10	1.285 (5)	C17—H17A	0.9300
N3—N4	1.387 (6)	C8—C9	1.483 (7)
N4—C11	1.299 (5)	C8—H8A	0.9700
N2—C9	1.349 (5)	C8—H8B	0.9700
N2—C10	1.381 (6)	C13—H13A	0.9300
N2—H2A	0.8600	C7—C6	1.469 (6)
C4—C5	1.361 (7)	C6—C1	1.383 (5)
C4—C3	1.382 (6)	C2—C3	1.335 (7)
C4—H4B	0.9300	C2—C1	1.388 (6)
C5—C6	1.367 (7)	C2—H2B	0.9300
C5—H5A	0.9300	C3—H3B	0.9300
C16—C15	1.359 (7)	C1—H1B	0.9300
			
C10—S1—C11	86.0 (2)	C16—C17—H17A	119.5
C7—N1—C8	119.6 (3)	N1—C8—C9	112.3 (3)
C7—N1—H1A	120.2	N1—C8—H8A	109.1
C8—N1—H1A	120.2	C9—C8—H8A	109.1
C10—N3—N4	110.7 (4)	N1—C8—H8B	109.1
C11—N4—N3	113.3 (3)	C9—C8—H8B	109.1
C9—N2—C10	126.3 (3)	H8A—C8—H8B	107.9
C9—N2—H2A	116.8	O2—C9—N2	121.9 (4)
C10—N2—H2A	116.8	O2—C9—C8	125.0 (4)
C5—C4—C3	118.3 (5)	N2—C9—C8	113.1 (3)
C5—C4—H4B	120.9	N4—C11—C12	123.3 (4)
C3—C4—H4B	120.9	N4—C11—S1	113.4 (3)
C4—C5—C6	122.2 (4)	C12—C11—S1	123.3 (3)
C4—C5—H5A	118.9	C12—C13—C14	120.4 (4)
C6—C5—H5A	118.9	C12—C13—H13A	119.8
N3—C10—N2	119.8 (4)	C14—C13—H13A	119.8
N3—C10—S1	116.5 (4)	O1—C7—N1	120.5 (4)
N2—C10—S1	123.6 (3)	O1—C7—C6	120.0 (4)
C15—C16—C17	118.0 (4)	N1—C7—C6	119.4 (3)
C15—C16—H16A	121.0	C5—C6—C1	118.3 (4)
C17—C16—H16A	121.0	C5—C6—C7	118.5 (3)
C16—C15—C14	122.6 (5)	C1—C6—C7	123.2 (4)
C16—C15—Br1	119.4 (3)	C3—C2—C1	120.2 (4)
C14—C15—Br1	118.0 (4)	C3—C2—H2B	119.9
C13—C12—C17	119.7 (4)	C1—C2—H2B	119.9
C13—C12—C11	117.6 (3)	C2—C3—C4	121.2 (4)
C17—C12—C11	122.6 (4)	C2—C3—H3B	119.4
C13—C14—C15	118.2 (4)	C4—C3—H3B	119.4
C13—C14—H14A	120.9	C6—C1—C2	119.8 (4)
C15—C14—H14A	120.9	C6—C1—H1B	120.1
C12—C17—C16	121.0 (4)	C2—C1—H1B	120.1
C12—C17—H17A	119.5		
			
C10—N3—N4—C11	−1.0 (5)	C13—C12—C11—N4	−0.9 (6)
C3—C4—C5—C6	−2.5 (8)	C17—C12—C11—N4	178.3 (4)
N4—N3—C10—N2	179.7 (4)	C13—C12—C11—S1	179.6 (3)
N4—N3—C10—S1	2.2 (5)	C17—C12—C11—S1	−1.2 (6)
C9—N2—C10—N3	179.2 (4)	C10—S1—C11—N4	1.5 (3)
C9—N2—C10—S1	−3.6 (6)	C10—S1—C11—C12	−178.9 (4)
C11—S1—C10—N3	−2.2 (4)	C17—C12—C13—C14	1.8 (6)
C11—S1—C10—N2	−179.5 (4)	C11—C12—C13—C14	−179.0 (4)
C17—C16—C15—C14	−1.5 (7)	C15—C14—C13—C12	−1.8 (7)
C17—C16—C15—Br1	−179.8 (3)	C8—N1—C7—O1	5.2 (6)
C16—C15—C14—C13	1.7 (7)	C8—N1—C7—C6	−176.1 (4)
Br1—C15—C14—C13	−179.9 (3)	C4—C5—C6—C1	1.8 (7)
C13—C12—C17—C16	−1.6 (6)	C4—C5—C6—C7	−178.7 (5)
C11—C12—C17—C16	179.2 (4)	O1—C7—C6—C5	17.2 (7)
C15—C16—C17—C12	1.4 (7)	N1—C7—C6—C5	−161.5 (4)
C7—N1—C8—C9	−159.0 (4)	O1—C7—C6—C1	−163.3 (4)
C10—N2—C9—O2	−2.3 (6)	N1—C7—C6—C1	18.0 (7)
C10—N2—C9—C8	−180.0 (4)	C1—C2—C3—C4	0.1 (8)
N1—C8—C9—O2	−0.2 (6)	C5—C4—C3—C2	1.6 (8)
N1—C8—C9—N2	177.4 (3)	C5—C6—C1—C2	0.0 (7)
N3—N4—C11—C12	179.8 (4)	C7—C6—C1—C2	−179.5 (4)
N3—N4—C11—S1	−0.6 (5)	C3—C2—C1—C6	−0.9 (8)

### Hydrogen-bond geometry (Å, °)

**Table:**

*D*—H···*A*	*D*—H	H···*A*	*D*···*A*	*D*—H···*A*
C16—H16A···O2^i^	0.93	2.49	3.400 (5)	168
N2—H2A···O1^ii^	0.86	1.99	2.835 (5)	167

Symmetry codes: (i) −*x*, −*y*+1, −*z*+1; (ii) −*x*+1, −*y*+1, −*z*.
